# A Microliter-Scale High-throughput Screening System with Quantum-Dot Nanoprobes for Amyloid-β Aggregation Inhibitors

**DOI:** 10.1371/journal.pone.0072992

**Published:** 2013-08-26

**Authors:** Yukako Ishigaki, Hiroyuki Tanaka, Hiroaki Akama, Toshiki Ogara, Koji Uwai, Kiyotaka Tokuraku

**Affiliations:** Division of Applied Sciences, Muroran Institute of Technology, Muroran, Japan; Glasgow University, United Kingdom

## Abstract

The aggregation of amyloid β protein (Aβ) is a key step in the pathogenesis of Alzheimer’s disease (AD), and therefore inhibitory substances for Aβ aggregation may have preventive and/or therapeutic potential for AD. Here we report a novel microliter-scale high-throughput screening system for Aβ aggregation inhibitors based on fluorescence microscopy-imaging technology with quantum-dot Nanoprobes. This screening system could be analyzed with a 5-µl sample volume when a 1536-well plate was used, and the inhibitory activity could be estimated as half-maximal effective concentrations (EC_50_). We attempted to comprehensively screen Aβ aggregation inhibitors from 52 spices using this system to assess whether this novel screening system is actually useful for screening inhibitors. Screening results indicate that approximately 90% of the ethanolic extracts from the spices showed inhibitory activity for Aβ aggregation. Interestingly, spices belonging to the *Lamiaceae*, the mint family, showed significantly higher activity than the average of tested spices. Furthermore, we tried to isolate the main inhibitory compound from *Satureja* *hortensis* , summer savory, a member of the *Lamiaceae*, using this system, and revealed that the main active compound was rosmarinic acid. These results demonstrate that this novel microliter-scale high-throughput screening system could be applied to the actual screening of Aβ aggregation inhibitors. Since this system can analyze at a microscopic scale, it is likely that further minimization of the system would easily be possible such as protein microarray technology.

## Introduction

Many neurodegenerative disorders such as Alzheimer’s disease (AD), Parkinson’s disease, prion disease, and Huntington’s disease are associated with the aggregation and deposition of misfolded proteins, the amyloids [1,2]. Since aggregates containing oligomers and fibrils show toxicity towards neuronal cells, amyloid aggregation inhibitors may be key compounds in the regulation of these amyloid diseases. In general, inhibitory activity against amyloid aggregation is assessed by spectrophotometric assays using amyloid β protein (Aβ)-binding dyes (e.g., thioflavin-T (ThT) and Congo red [3,4,5]) or by direct observation of aggregates using transmission electronic microscopy (TEM) [6,7,8] and/or atomic force microscopy (AFM) [9,10]. However, dye-binding assays that use ThT and Congo red to evaluate these inhibitory effects could be prone to false positive effects because fluorescence intensities of these dyes may be influenced by the inner filter effects of contaminating compounds and/or the inhibitors themselves [11]. Moreover, there is a possibility of competition for binding between the dyes and inhibitors to amyloid fibrils. These problems are significant when screening novel active compounds. On the other hand, direct observation by TEM and AFM with fixation and washing processes is unsuitable for quantification and high-throughput analysis. Therefore, we have been attempting to develop a novel high-throughput screening system for amyloid aggregation inhibitors.

Recently we successfully developed real-time imaging and quantification of Aβ_42_ aggregation using quantum-dot (QD)-labeled Aβ_40_ (QDAβ) [12]. In that study, we showed that QDAβ, which had a binding ratio (Aβ_40_/QD) of 6, was incorporated into Aβ fibrils with a similar efficiency as unlabeled Aβ_42_ when 0.1-0.01% QDAβ was mixed with unlabeled Aβ_42_ [12]. The time to reach the steady state was similar to that of intact Aβ_42_ measured from turbidity measurements [13]. Moreover, there are only small amounts (0.1-0.01%) QDAβ relative to unlabeled Aβ_42_, suggesting that this imaging technology can monitor the physiological aggregation of intact Aβ_42_. Since the amount of aggregates decreased in the presence of anti-Aβ antibody, we proposed that this imaging technology can be applied to the microliter-scale screening of inhibitory substances for Aβ_42_ aggregation [12].

To attain a microliter-scale and high-throughput screening system, in this study, we tried to optimize the observation methods and to develop a simple quantification method from fluorescence microscopic images. Furthermore, in order to confirm whether this microliter-scale high-throughput screening system can actually be used to screen Aβ aggregation inhibitors, we attempted to screen inhibitory substances for Aβ aggregation from 52 dried spices that include various plant species that are readily commercially available.

## Results

### Methodology of the microliter-scale high-throughput screening system for Aβ aggregation inhibitors

In this study, we used a 1536-well plate. When a 96-well plate was used in a recent study [12], at least 40 µl of sample volume was necessary to prevent the sample from drying. The use of a 1536-well plate enabled the observation of aggregates using a 5-µl sample volume without the drying side-effect. To develop an image of Aβ aggregates, in this study, we used Qdot655 and a customized the fluorescence filter block whose excitation filter was adjusted from 425 nm to 470 nm to attenuate the inner filter effect by substances in samples. For example, maximum absorption of curcumin, which is a known Aβ aggregation inhibitor, is 426 nm [11,14], so that fluorescence intensity may be influenced if an excitation filter of 425 nm is used. Since Qdot655 has a higher extinction coefficient than Qdot525, which we used recently [12], over a wide range of excitation wavelengths, Qdot655 fluorescent light could be sufficiently detected by a general CCD camera, even when the excitation wavelength was 470 nm.

**Figure 1:**
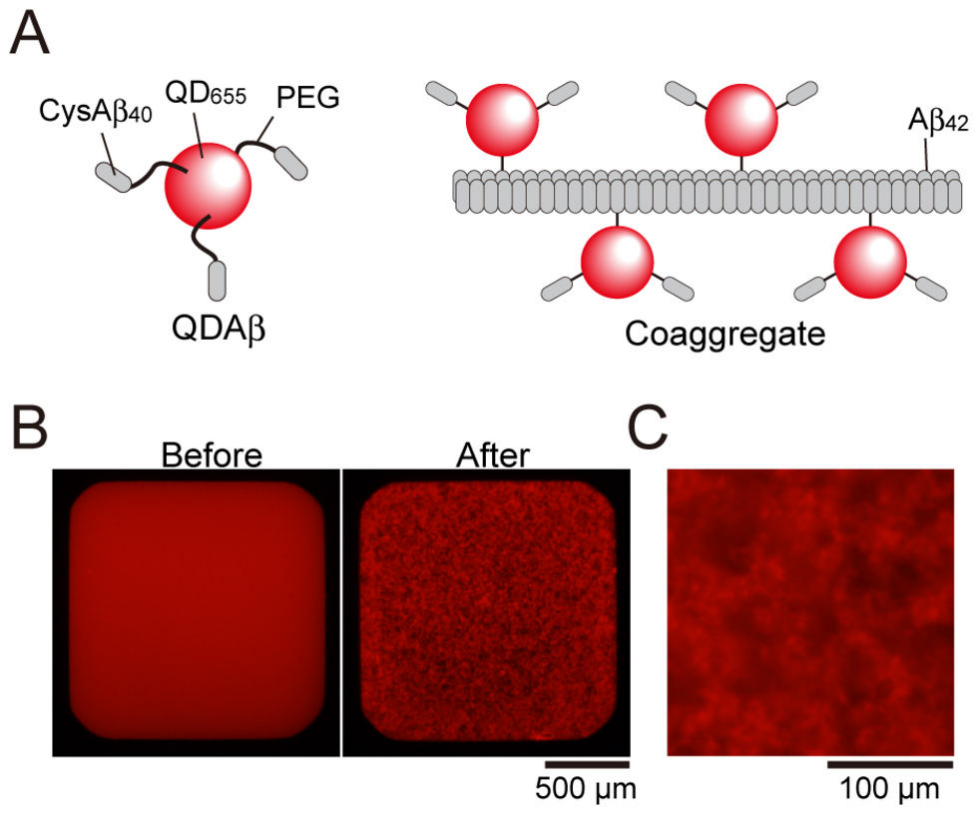
Imaging of Aβ_42_ aggregation using a QDAβ nanoprobe. (A) QDAβ was prepared by crosslinking CysAβ_40_ and amino (PEG) Qdot655 according to our recent study [12] (left). QDAβ coaggregated with unlabeled Aβ_42_, and the Aβ_42_ fibrils that formed could be visualized under fluorescence microscopy (right). (B) 30 nM QDAβ and 30 µM unlabeled Aβ_42_ was incubated at 37 ^°^C for 24 h in a 1536-well plate, and was observed using an inverted fluorescence microscope using a 4x objective. Left and right panels show before and after incubation, respectively. (C) Magnified image of the aggregates observed using a 10× objective.

**Figure 2:**
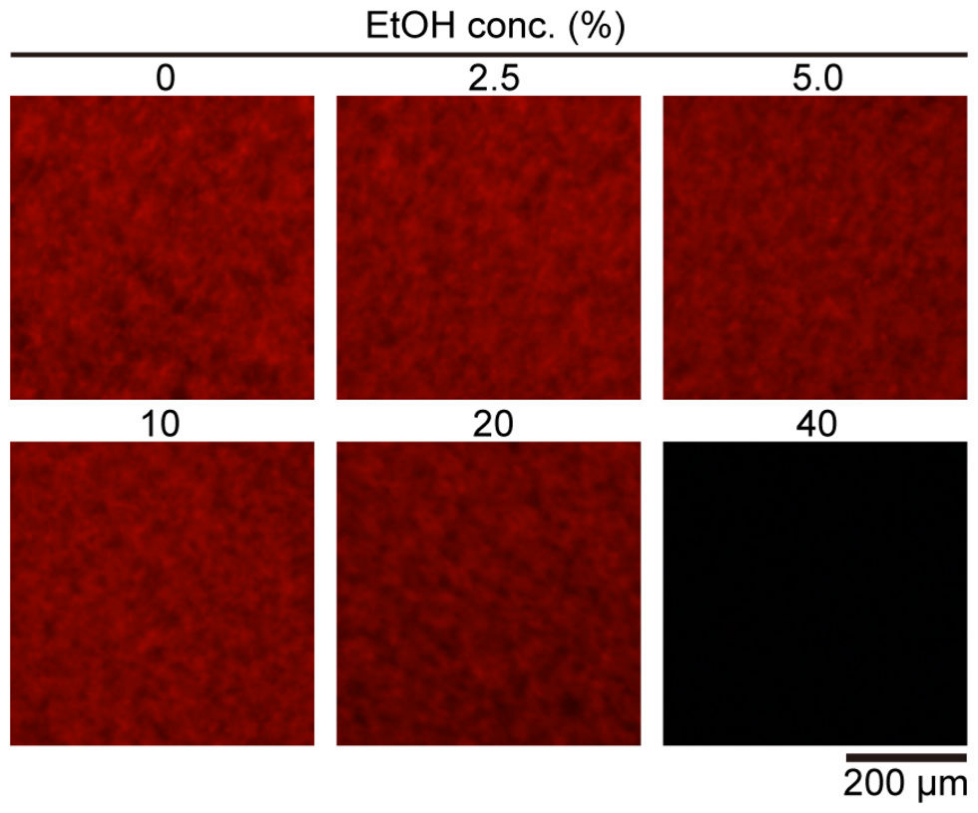
Effect of EtOH on Aβ_42_ aggregation. 30 nM QDAβ and 30 µM Aβ_42_ were mixed in 1xPBS, 3% DMSO containing 0, 2.5, 5, 10, 20, or 40% EtOH, each sample was incubated at 37 ^°^C for 24 h in a 1536-well plate. The wells were observed using an inverted fluorescence microscope using a 4x objective. The images show 200 × 200 pixels in the center of each well.

When 30 nM of QDAβ and 30 µM of Aβ_42_ were incubated in a 1536-well plate at 37 ^°^C for 24 h, their aggregates were visualized, as reported in our recent study [12] (Figure 1). First, we examined the effect of EtOH on Aβ aggregation because we used EtOH extracts of spices in the next screening step (Figure 2). 40% EtOH immediately induced amorphous aggregates, which are not fibrils, of QDAβ and Aβ_42_, and the amorphous aggregates were eliminated by centrifugation at 10,000 x*g* for 2 min at 4 ^°^C before incubation. Consequently, no fluorescence was observed in the sample in the presence of 40% EtOH (Figure 2, 40%). When the sample was incubated with 20% EtOH, it seemed that the aggregates were slightly reduced (Figure 2, 20%). The data showed that the aggregation was not affected by 10% EtOH (Figure 2, 10%), so we performed all experiments in the presence of 5% EtOH in this study.

**Figure 3:**
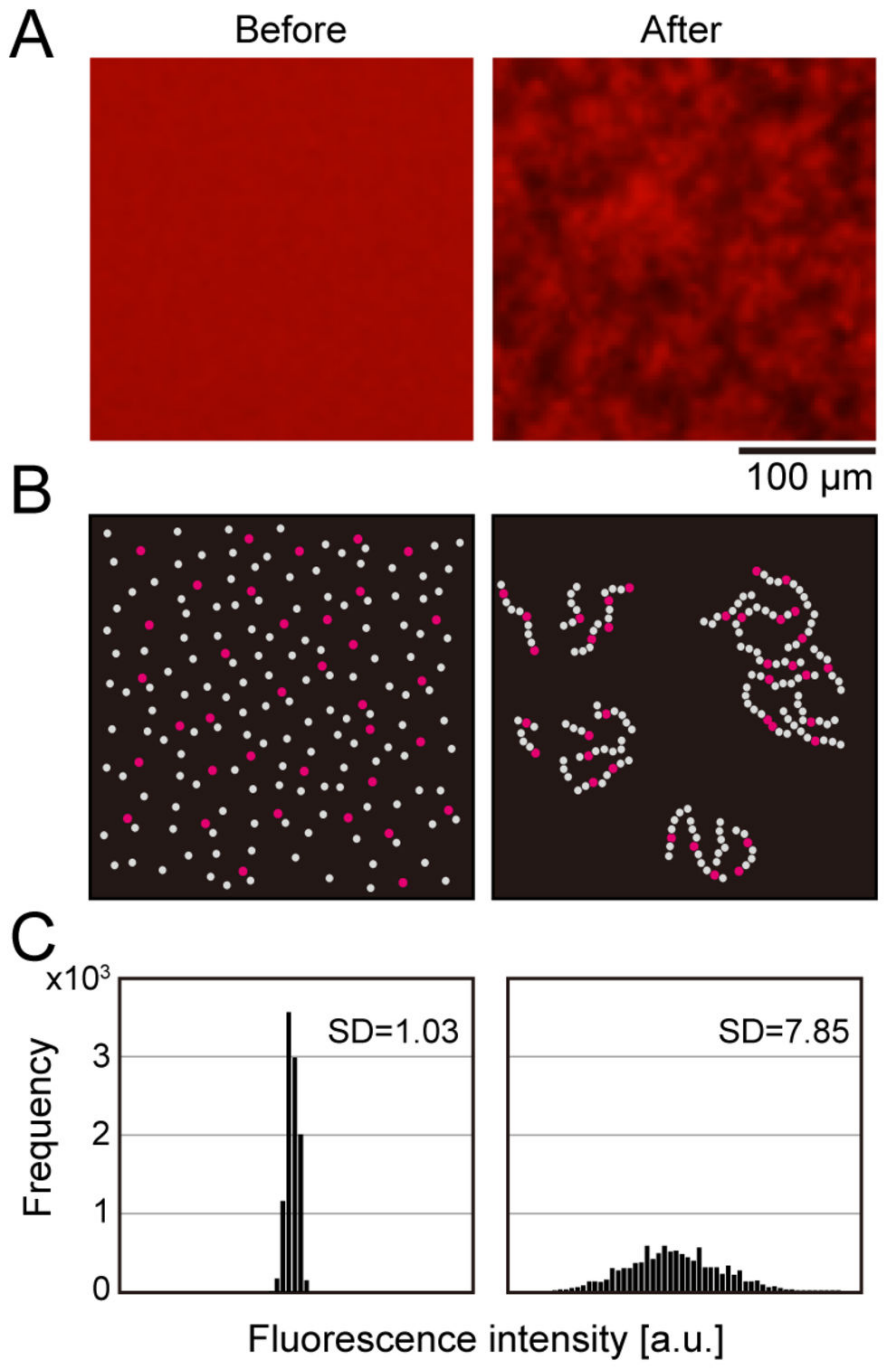
Correlation between Aβ aggregation and variations of fluorescence intensity. (A) Magnified images of center region (100 × 100 pixel) in fluorescence micrographs of QDAβ- Aβ_42_ coaggregates before (left) and after (right) incubation (Figure 1B). (B) Schematic illustrations of the distribution of QDAβ (red) and Aβ_42_ (gray) molecules before (left) and after (right) incubation of samples. QDAβ molecules are diffused in the sample solution before incubation (left), and QDAβ molecules are inserted in Aβ_42_ fibrils after incubation (right). (C) The histograms of fluorescence intensities of 10,000 pixels (100 × 100 pixel) before (left) and after (right) incubation of samples.

Next, we explored a way to quantify the amount of aggregates from the fluorescence microscopy images. We had already reported that the volume of Aβ aggregates can be estimated from 3D-images by confocal fluorescence microscopy [12]. However, the time taken for 3D-image acquisition is longer than that for 2D-image acquisition, and data size of the 3D-image is also larger than that of the 2D-image because one 3D-image reconstruction requires several dozen 2D-images. Therefore, we considered a simple quantification method from one 2D-image. Before incubation, QDAβ molecules were dispersed in a sample solution, so that the fluorescence micrograph showed a uniform red color (Figure 3A; left panel). After incubation, Aβ aggregates that were visualized by QDAβ were observed on the well bottom (Figure 3A; right panel). This aggregation resulted inhomogeneous distribution of fluorescence intensity in micrographs (Figure 3B). The histogram of fluorescence intensities of 10,000 pixels (100 × 100 pixels) prior to sample incubation was narrow but that after sample incubation was broad, and the standard deviation (SD) of post-incubation samples was larger than that of pre-incubation samples (Figure 3C), suggesting that SD values correlated with the amount of Aβ aggregates. That is, as Aβ aggregation progressed, the variability of fluorescence intensities of each pixel increased, and standard deviation (SD) values also increased.

**Figure 4:**
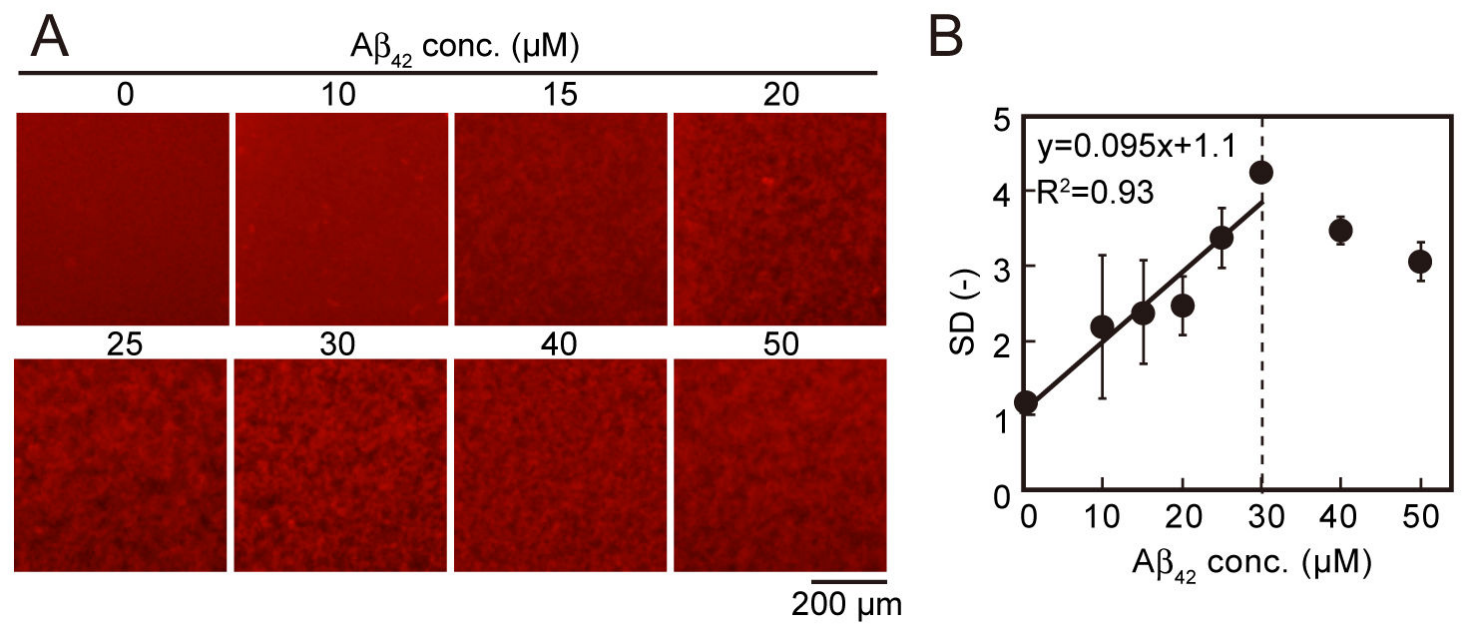
Concentration-dependent Aβ aggregation. (A) Various concentrations of Aβ_42_ and 30 nM QDAβ were incubated in a 1536-well plate at 37 ^°^C for 24 h. Each well was observed using an inverted fluorescence microscope using a 4x objective. (B) Variations of fluorescence intensities of 10,000 pixels (100 × 100 pixel) in the center region of micrographs were estimated as SD values, the mean values were plotted against the concentrations of added Aβ_42_. A linear equation and R^2^ in B were determined using the data of less than 30 µM of Aβ concentrations. Error bars represent ±SDs of the mean values of fluorescence intensities (n=3 separate experiments).

**Figure 5:**
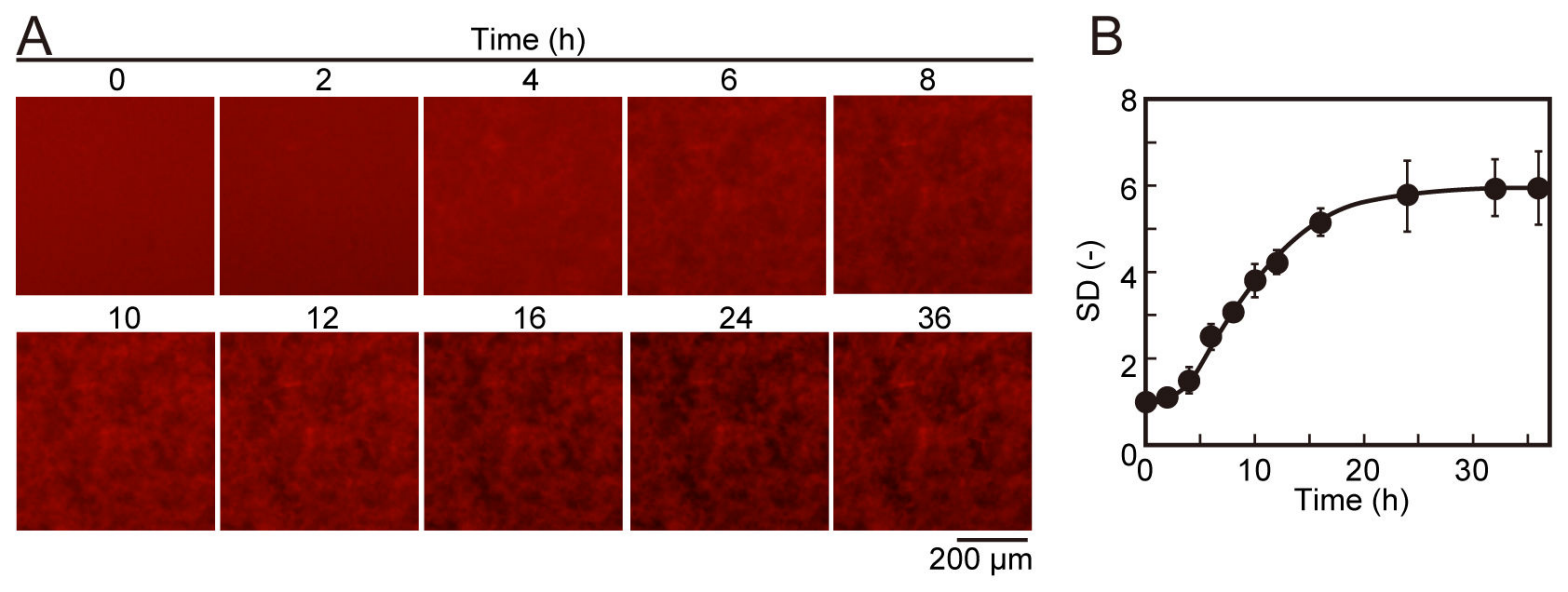
Time-dependent Aβ aggregation. (A) 30 nM QDAβ and 30 µM Aβ_42_ were incubated in a 1536-well plate at 37 ^°^C, and observed over time by an inverted fluorescence microscope using a 4x objective. All images show the same field of a well. (B) Variations of fluorescence intensities of 10,000 pixels (100 × 100 pixel) in the center region of micrographs were estimated as SD values, the mean values were plotted against incubation time periods. Error bars represent ±SDs of the mean values of fluorescence intensities (n=3 separate experiments).

To confirm the correlation between the amount of Aβ aggregates and SD values, we attempted a concentration-dependent assay (Figure 4). 30 nM QDAβ and various concentrations of Aβ_42_ were incubated in a 1536-well plate and the images (Figure 4A) were analyzed using ImageJ software (NIH). When the Aβ_42_ concentration was low (10–25 µM), diffused QDAβ molecules were observed in the intervening space between Aβ aggregates, so that the intervening spaces remained red due to QDAβ (Fig. 4A). When Aβ_42_ concentration was around 30 µM, almost all QDAβ molecules were inserted into Aβ_42_ fibrils, so that the intervening spaces were darker than that of the low Aβ_42_ concentrations (Fig. 4A). When Aβ_42_ concentration was high (40–50 nM), accumulated Aβ aggregates at the well bottom were thick, so that out-of-focus aggregates were imaged in the fluorescence micrographs, causing a blurred image (Fig. 4A). The thickness of Aβ aggregates on the well bottom was about 50 µm when 100 µM Aβ_42_ was incubated in a 96-well plate [12]. The depth of sample solutions in the 96-well plate and in the 1536-well plate is the same (2 mm), suggesting that the thickness of aggregates might be about 15 µm in the presence of 30 µM Aβ_42_. Since the depth of focus of the microscopic system used in this study was approximately 14 µm it is likely that the gradual decrease of SD values (Figure 4B) over 30 µM of Aβ_42_ concentration was caused by an increase of out-of-focus aggregates. Linear regression analysis showed that the SD values from the fluorescence micrographs increased in a concentration-dependent manner at less than 30 µM of Aβ_42_ (R^2^ = 0.93) (Figure 4B). In this study, therefore, we screened using 30 µM Aβ_42_ in the following steps. Furthermore, we confirmed whether time-dependent SD values increased as Aβ aggregation progressed. The micrographs showed time-dependent aggregation (Figure 5A), and the SD values also increased in a time-dependent manner (Figure 5B). The time-dependent graph (Figure 5B) showed a typical kinetic curve for amyloid aggregation which consisted of time lag, growth, and steady state phases, similar to recent 3D volume data by confocal microscopy [12]. Although we have no conclusive evidence whether SD values increase in direct proportion to the amount of Aβ_42_ aggregates, the concentration- (Figure 4B) and time-dependent (Figure 5B) SD data suggests that the SD values could be used as an approximate indicator of Aβ aggregates. Since the time-dependent data revealed that the aggregation reached a plateau around 24 h (Figure 5B), incubation time was fixed at 24 h in the following screening steps.

**Figure 6:**
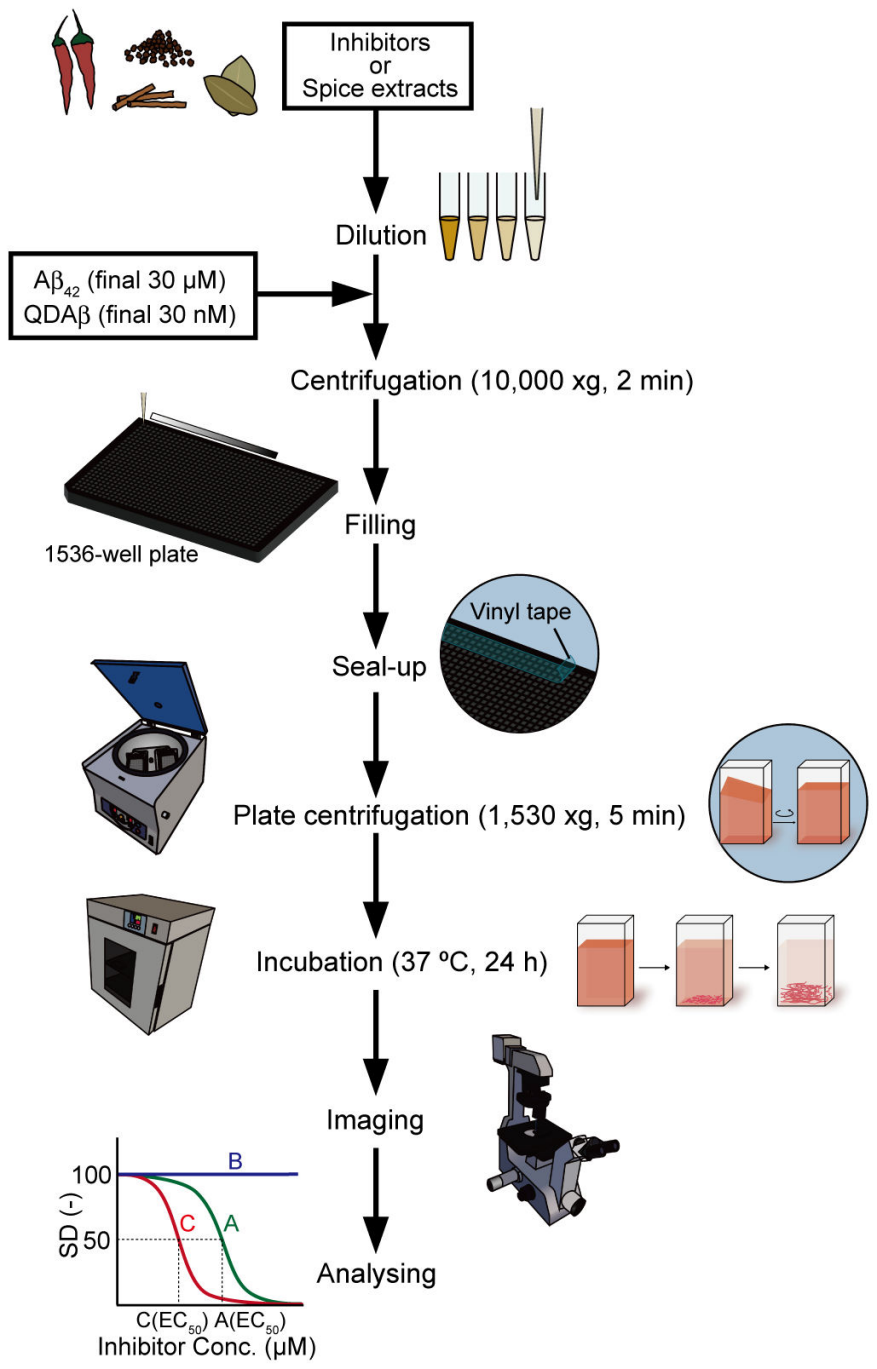
A flow diagram of the microliter-scale high-throughput screening system. Various concentrations of known inhibitors or spice extracts were incubated with 30 µM Aβ_42_ and 30 nM QDAβ in a 1536-well plate, and incubated to induce the aggregation of Aβ_42_. Aggregates of Aβ_42_ and QDAβ were imaged by fluorescence microscopy, and EC_50_ of the inhibitory activities were estimated from the fluorescence micrograph data A more detailed method is mentioned in the experimental section.

**Figure 7:**
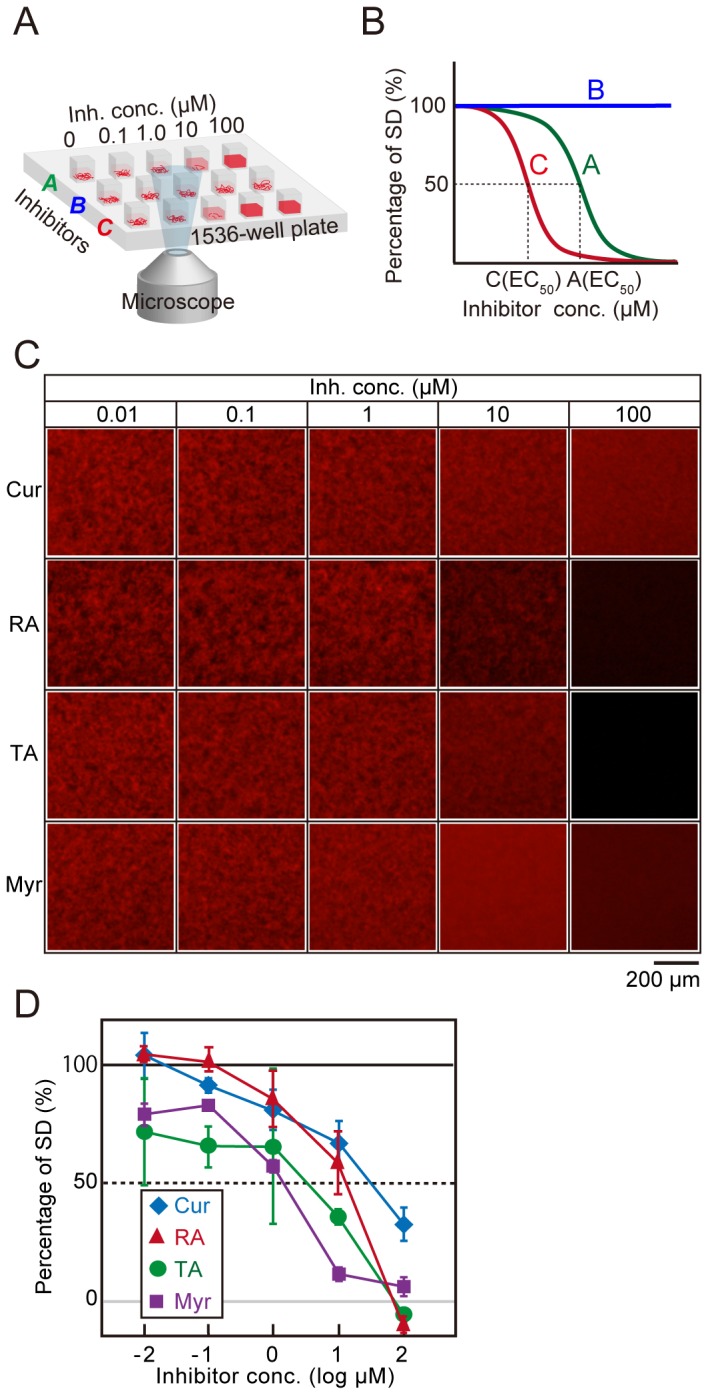
Estimation of EC_50_ by the microliter-scale high-throughput screening system. (A) A schematic illustration of a microliter-scale high-throughput screening system for three samples. (B) The concept of estimation of EC_50_ values from inhibition curves that are a plotted percentage of SD versus concentrations of inhibitors. The EC_50_ of sample A is higher than that of sample C, and sample B did not inhibit Aβ aggregation. (C and D) Estimations of EC_50_ of well-known inhibitors, curcumin (Cur), rosmarinic acid (RA), tannic acid (TA), and myricetin (Myr). 30 nM QDAβ and 30 µM Aβ_42_ was incubated with various concentrations of the four inhibitors at 37 ^°^C for 24 h (C). The SD values from the fluorescence images plotted against several concentrations of inhibitors (D). Error bars represent ±SDs of the mean values from fluorescence intensities (n=3 separate experiments). EC_50_ values of Cur, RA, TA, and Myr were 31 ± 16, 11 ± 2, 1.8 ± 1.5, and 1.0 ± 0.3 µM, respectively.

On the basis of the above results, we set up the microliter-scale high-throughput screening system as mentioned in the Experimental section and Figure 6. Simply put, various concentrations of candidate inhibitors were incubated with 30 nM QDAβ and 30 µM Aβ_42_ in a 1536-well plate (Figures 6 and 7A) at 37 ^°^C for 24 h, and SD values of fluorescence intensity in each micrograph were analyzed by ImageJ software. The half-maximal effective concentration (EC_50_) values of inhibitors could be estimated from the inhibition curves (Figure 7B). Figure 7D shows the actual inhibition curves of well-known Aβ aggregation inhibitors that were determined from the SD values of fluorescence micrograph data (Figure 7C). The EC_50_ could be estimated from the inhibition curves using an EC_50_ shift by the Prism global fitting program (GraphPad software).

### Comprehensive screening of Aβ aggregation inhibitors from 52 spices

**Figure 8:**
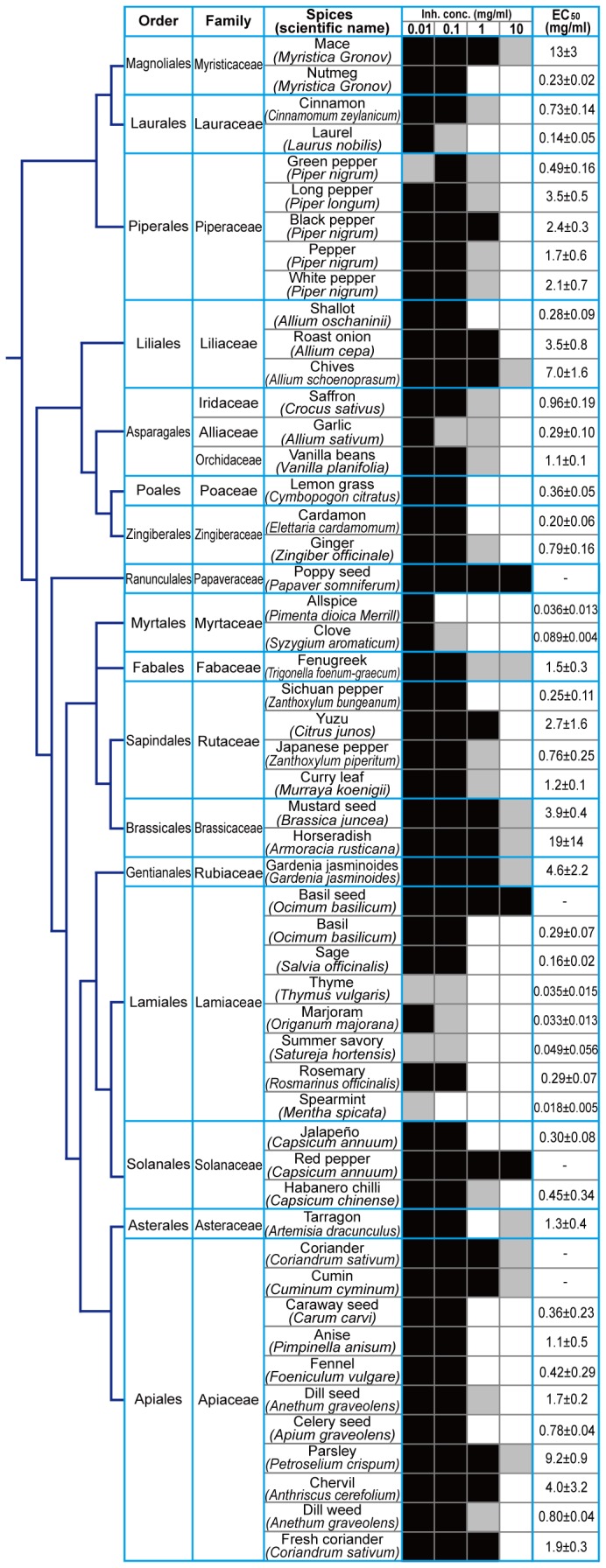
Estimation of EC_50_ values of EtOH extracts from 52 spices using the microliter-scale high-throughput screening system. Screening was applied to examine the EtOH extracts of dried spices. The ‘black’, ‘gray’, and ‘white’ cells indicate ‘not inhibited (SD ≥ 80%)’, ‘partially inhibited (80% > SD > 20%)’, and ‘completely inhibited (20% ≥ SD)’ wells, respectively. The percentage of SD was defined as SD values before and after incubation of control samples (0% and 100%, respectively). EC_50_ values were estimated from dose-dependent inhibition curves (n=3 separate experiments). Spices were aligned using the Angiosperm Phylogeny Group classification (APG III) [25].

Since we confirmed that the microliter-scale high-throughput screening system can be applied to estimate the EC_50_ of Aβ aggregation inhibitors, we then tried to actually screen the EtOH extracts of 52 dried spices belonging to 19 plant families (Figure 8). The results revealed that many spices (about 90%) had inhibitory activity for Aβ aggregation when the extracts were added at a high concentration (~10 mg). On the other hand, there are five spices that did not show inhibitory activity (Figure 8), demonstrating that this high degree of inhibitory success was not caused by methodological problems of this screening system. That is, it is likely that the “not effective” spices were comparable to negative controls of this screening system. The spices belonging to the *Lamiaceae* and *Myrtaceae* families showed significantly higher inhibitory activity against Aβ aggregation than other family members. Especially, thyme ( *Thymus* *vulgaris* ) (EC_50_ = 0.035 ± 0.015 mg/ml), summer savory ( *Satureja* *hortensis* ) (EC_50_ = 0.049 ± 0.056 mg/ml), and spearmint ( *Mentha* *spicata* ) (EC_50_ = 0.018 ± 0.005 mg/ml), all belonging to the *Lamiaceae* (mint) family, affected Aβ aggregation at 0.01 mg/ml (Figure 8, gray cells on 0.01 mg/ml line). Although green pepper ( *Piper* *nigrum* ) also affected SD values at 0.01 mg/ml it is likely that the result was an error because the effect was not observed at 0.1 mg/ml of extract concentration. All spices that did not show inhibitory activity (Figure 8, poppy seed ( *Papaver* *somniferum* ), basil seed ( *Ocimum* *basilicum* ), red pepper ( *Capsium* *annuum* ), coriander ( *Coriandrum* *sativum* ), and cumin ( *Cuminum* *cyminum* )) were spices originating from seeds while only red pepper contained fruit.

**Figure 9:**
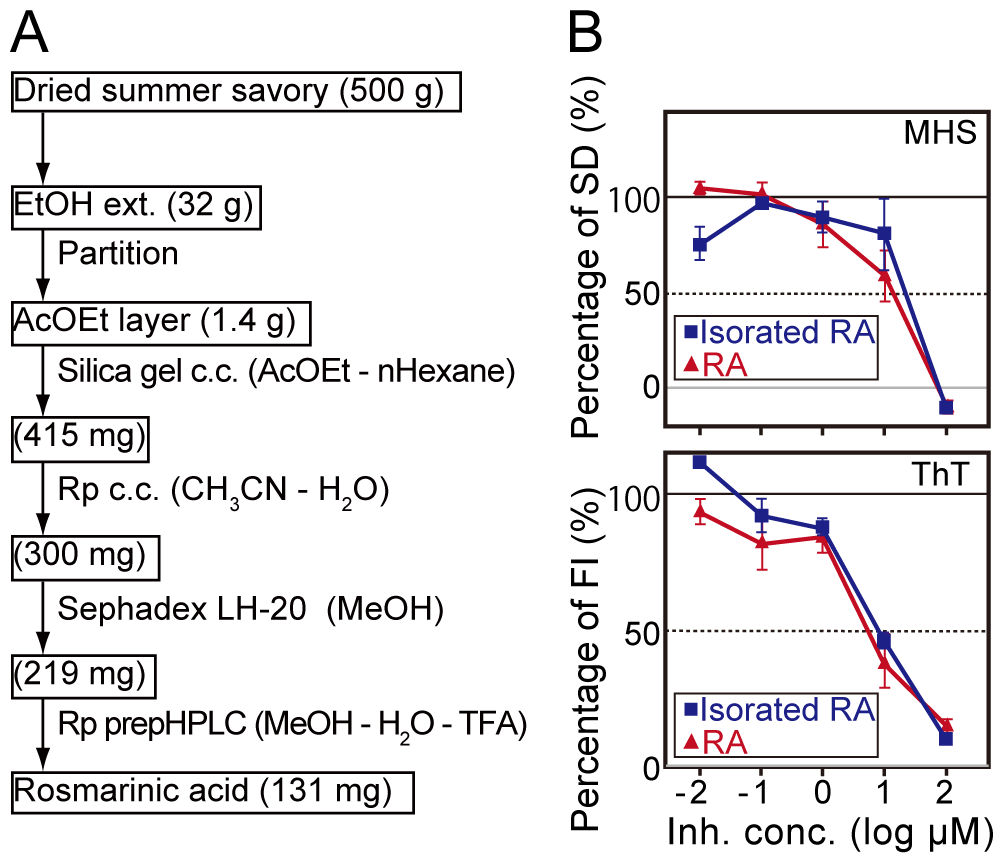
Isolation and identification of active compound from EtOH extract of summer savory. (A) A flow diagram of isolation steps. (B) Inhibition curves of isolated RA from summer savory (squares) and standard RA (triangles) were determined by the microliter-scale high-throughput screening (MHS) system (B, top) and the ThT assay (B, bottom). Vertical axes of the MHS system and the ThT assay are the percentage of average SD values and the percentage of average fluorescence intensity (FI) values, respectively. The EC_50_ values of isolated RA and standard RA determined by the MHS system were 9.6 ± 0.1 and 11 ± 2 µM, respectively. In contrast to that, the EC_50_ values of isolated RA and standard RA determined by ThT assay were 8.6 ± 0.8 and 6.3 ± 1.5 µM, respectively. Error bars represent ±SDs (n=3 separate experiments).

We then tried to isolate the main active substance from summer savory, which was one of the spices that showed the highest inhibitory activity, under the guidance of the inhibitory effect (EC_50_) against Aβ aggregation determined by the microliter-scale high-throughput screening system (Figure 9). The ethanolic extract (32 g) of dried summer savory (500 g) was successively partitioned between CHCl_3_, AcOEt, *n*-BuOH and H_2_O (Figure 9A). The AcOEt layer, the best active fraction, was applied to silica gel column chromatography, reverse phase column chromatography, gel filtration, and reverse phase HPLC to give a single compound (131 mg) (Figure 9A), which had ESI-MS, NMR, and IR spectra identical to rosmarinic acid (RA). The inhibitory activities of the isolated RA and standard RA analyzed by the microliter-scale high-throughput screening system (Figure 9B, top) and the ThT assay (Figure 9B, bottom) were similar, supporting the conclusion that the isolated substance was RA.

## Discussion

In this study, we successfully developed a novel microliter-scale high-throughput screening system with QD for Aβ aggregation inhibitors (Figure 6). The comprehensive screening of Aβ aggregation inhibitors for 52 spices using the screening system revealed that almost all spices had inhibitory activity for Aβ aggregation (Figure 8). Masuda et al. [15] reported that many polyphenols inhibit the aggregation of Aβ and α-synuclein, which is another amyloid. Polyphenols are natural substances present in many plants that are consistent with our result showing that almost all of the 52 spices tested had inhibitory activity. Furthermore, we demonstrated that the *Lamiaceae* family, except for basil seed, showed higher inhibitory activity against Aβ aggregation than all other plant families (Figure 8), and that RA was the main active substance of summer savory (Figure 9). These results suggest that this system could be applied to the actual screening of Aβ aggregation inhibitors.

RA was previously reported to inhibit Aβ aggregation *in vitro* [16] and Aβ deposition of RA-treated AD model transgenic mice decreased significantly in the brain [17]. Moreover, it was reported that RA prevented Aβ oligomerization and synaptic dysfunction by site-specific binding [18]. Interestingly, *Salvia* *officinalis* (sage) and *Melissa* *officinalis* (lemon balm), also belonging to the *Lamiaceae* family, had already been reported to have memory-improving properties in old European reference books hundreds of years ago [19]. It is possible that the effect involves the inhibitory activity of Aβ aggregation of RA, which is found in members of the *Lamiaceae* family [20]. We also found that some fractions, which were separate fractions in the isolation process from summer savory (Figure 9), showed higher inhibitory activity than RA, but that the content was smaller than RA, thus, they were not major active fractions. Since this screening system can evaluate a small amount of sample, we are now trying to isolate the highly active compounds from the fractions (data to be published soon).

The EC_50_ values that were determined by the microliter-scale high-throughput screening system (Figures 7 and 9) showed a tendency to be higher than the values that were determined by ThT fluorescent intensities [15,16,21]. As mentioned above, it has been reported that a dye-binding assay using ThT has the potential to show false positive effects such as inner filter effects [11]. In contrast, since this screening system adopts a longer excitation spectrum and quantification from variability data of fluorescence intensity, it is likely that the inner filter effects are smaller than those of the ThT assay.

Recently, potent amyloidogenicity and pathogenicity of Aβ_43_ was reported [22]. The microliter-scale high-throughput screening system can be applied easily by changing unlabeled Aβ_42_ peptide to Aβ_43_ peptide. Furthermore, this system could be applied to screening inhibitors for the aggregation of other amyloid peptides such as α-cynuclein, huntingtin, and prion. In addition, it is likely that this principle can be used to screen inhibitors or promoters for polymerization of normal filament-forming protein such as actin and microtubules.

The toxicity of soluble oligomers is a trigger for neurodegenerative diseases [23,24], indicating that Aβ oligomerization inhibitors are also important for preventive and/or therapeutic agent. The growth of aggregates occurred after the nucleation step, i.e., the oligomerization step, suggesting that oligomerization inhibitors also affect the amount of Aβ aggregates in the same incubation period. In fact, it was recently demonstrated that RA and Myr blocked Aβ oligomerization [18]. Therefore, it is reasonable to assume that oligomerization inhibitors can be screened as a second screening after the first comprehensive screening of Aβ aggregation inhibitors using this microliter-scale high-throughput screening system. We have already proposed a quantification method for oligomerization using QDAβ in a recent report [12]. It is likely that the combination of these methods would be useful to screen inhibitory substances for each aggregation process.

Although this system could quantify Aβ aggregation with only a 5-µl sample volume when a 1536-well plate was used, further minimization is possible such as protein microarray technology because the microscopic sample size is sufficient for data analysis in this system. This minimization would allow for more high-throughput screening. In addition, it is also one of the biggest advantages that this system does not need any handling processes, such as sample transfer by pipetting, after aggregation. Amyloid aggregates are hydrophobic and stick to plastic surfaces, and therefore are easy to adhere to, including to micropipette tips, microtubes, and multi-well plates. This can be a cause of a serious experimental error in the quantification of aggregates.

Although many compounds have shown inhibitory activity against Aβ aggregation [15], the mechanism of inhibition remains unknown. Since this microliter-scale high-throughput screening system can be applied to comprehensive screening, the isolation of Aβ aggregation inhibitors from natural sources and chemical libraries may be accelerated from now on. It is expected that a quantitative structure-activity relationship study, which would use many types of inhibitors that can be identified by this screening system, would demonstrate an inhibition mechanism. Such information would be helpful in the molecular design of inhibitors in drug discovery. Our hope is that this novel technology may be a powerful tool for the analysis of protein aggregation/assembly.

## Experimental Section

### Materials

Human amyloid peptides of Aβ_42_ (4349-v, Peptide Institute) and Cys-conjugated Aβ_40_ (CysAβ) (23519, Anaspec) were dissolved at a concentration of 1 mg/ml in 1,1,1,3,3,3-hexafluoro-2-propanol (083-04231, Wako Pure Chemical Industries), incubated at room temperature for 1 h, and sonicated for 10 min. The aliquots were dried down in microcentrifuge tubes and the dried films were stored at -20 ^°^C. N-(6-maleimidocaproyloxy) sulfosuccinimide ester (Sulfo-EMCS) (22307, Pierce) was stored at 4 ^°^C and dissolved in 8.1 mM Na_2_HPO_4_, 1.5 mM KH_2_PO_4_, 2.7 mM KCl, 137 mM NaCl, pH 7.4 (PBS) immediately before use. Amine-derivatized polyethylene glycol (PEG)-conjugated Qdot655 (QD-PEG-NH_2_) (Q21521MP, Life Technologies) was stored at 4 ^°^C. Known standard inhibitors, curcumin (038-04921), tannic acid (201-06332), myricetin (137-16791), and rosmarinic acid (182-02691), were purchased from Wako Pure Chemical Industries. All dried spices were purchased from S&B Foods Inc. All other chemicals were of reagent grade.

### Preparation of QDAβ

QDAβ was prepared according to our recent report [12] and a book (K. Tokuraku & T. Ikezu, Imaging of amyloid-β aggregation using a novel quantum dot nanoprobe and its advanced applications, Bionanoimaging-Insights into protein misfolding & aggregation, edited by Yuri Lyubchenko & Vladimir Uversky, Elsevier, in press). 10 µM QD-PEG-NH_2_ was first reacted with 1 mM sulfo-EMCS in PBS for 1 h at 20 ^°^C. After quenching and elimination of unreacted sulfo-EMSC, the QD-PEG-NH_2_-bound sulfo-EMCS was reacted with 100 µM CysAβ in PBS containing 5 mM EDTA for 1 h at 20 ^°^C. The labeling ratio of Aβ to QD can be controlled by the initial concentration of the added CysAβ [12]. In this condition, the labeling ratio (Aβ/QD) was estimated as being approximately 6 [12]. The concentrations of QDAβ were determined at an absorbance of 504 nm according to instruction manual of Life Technologies.

### Microliter-scale high-throughput screening system

Dried Aβ_42_ films were completely dissolved in 1 mM DMSO by pipetting for at least 15 min with the tip of a micropipette pressed on the bottom of a microtube. QDAβ and the dissolved Aβ_42_ were mixed in 1x PBS at final concentrations of 60 nM and 60 µM, respectively. 3 µl of the mixture of QDAβ and Aβ_42_ and 3 µl of various concentrations of inhibitors or spice extracts, which were diluted in 1x PBS containing 10% EtOH, were mixed (final condition was various concentrations of inhibitors or spice extracts, 30 nM QDAβ, 30 µM Aβ_42_, 1x PBS, 5% EtOH, 3% DMSO), and centrifuged at 10,000 x*g* for 2 min at 4 ^°^C to eliminate any insoluble dusts. 5 µl of the supernatants were transferred into each well of a 1536-well plate (782096, Greiner), and were tightly sealed with vinyl tapes to prevent the drying of sample solutions in wells. The 1536-well plate with sample solutions was centrifuged by a multi-well plate centrifuge (PlateSpin II, Kubota) at 1,530 x*g* for 5 min at room temperature to flatten the surface of sample solutions, and incubated at 37 ^°^C for 24 h in an air incubator (SIB-35, Sansyo). The 1536-well plate was observed by an inverted fluorescence microscope (Diaphot 300, Nikon) equipped with a color CCD camera (DP72, Olympus). QDAβ was imaged using a customized Qdot655 filter set (XF305-1, Omega optical; excitation 425DF45, dichroic 475DCLP, emission 655DF20) in such a way that the excitation filter was replaced by another filter (Ex 450-490 nm, Nikon) in order to reduce the interfering QD excitation by components in spice extracts and by the inhibitory compound itself.

### Estimation of EC_50_ from fluorescence microscopy images

To estimate the half-maximal effective concentration (EC_50_) that would inhibit Aβ aggregation, we used fluorescence micrograph data that was taken using a 4x objective lens with a customized Qdot655 filter set. Fluorescence intensities of 10,000 pixels (100 × 100 pixels: 280 × 280 µm) in the central region of each well and the SD values were measured by ImageJ software (NIH). The means and ±SDs of the mean values were calculated from three independent experiments. We defined EC_50_ as the inhibitor concentration when the SD value showed half of the maximum (after incubation) and minimum (before incubation) SDs of control sample without inhibitor. In particular, the inhibition curves (e.g. Figure 1G) were analyzed with Prism (GraphPad software) using an EC_50_ shift by global fitting (Asymmetric sigmoidal, 5 parameter logistic).

### Estimation of EC_50_ from ThT fluorescence intensities

EC_50_ for Aβ aggregation by ThT fluorescence was determined according to the method of Levine [4] modified in our laboratory. ThT (202-01002, Wako Pure Chemical Industries) was dissolved in water at 100 µM for 1 day at room temperature under constant stirring and shielded from light. The 100 µM ThT solution was diluted immediately before use in 50 mM glycine/KOH buffer (pH 8.5) to a final concentration of 5 µM. 30 µM Aβ_42_ and various concentrations of inhibitors were incubated in 1x PBS containing 5% EtOH and 3% DMSO for 24 h at 37^°^C. 10 µl of the incubated samples were added to 190 µl of 5 µM ThT solution, and the fluorescence intensity was measured at 490 nm after excitation at 455 nm in micro fluorimeter cells (FM20B, GL Science) using a fluorescence spectrophotometer (F-4500, Hitachi). EC_50_ was estimated from an inhibition curve using the EC_50_ shift by Prism global fitting software.

### Preparation of EtOH extracts from 52 dried spices

One g of each dried spice was extracted with 5 ml of EtOH at room temperature for 7 days. The EtOH solution was filtered and concentrated *in vacuo*.

### Isolation of active compound from summer savory

Dried summer savory (500 g) was extracted with 2.5 l of EtOH at room temperature for 7 days. The EtOH extract was filtered and concentrated *in vacuo* to yield a residue (32 g) that was partitioned between CHCl_3_, AcOEt, *n*-BuOH and H_2_O. Column chromatography of the concentrated AcOEt layer (1.41 g) on silica gel (100 g) (*n*-hexane-AcOEt (6:4, 5:5, 4:6, 3:7, 2: 8 v/v), AcOEt, AcOEt-MeOH (9:1, 7: 3 v/v) and MeOH) yielded 10 fractions (fractions A to J). Fraction E (0.44 g), which was eluted with *n*-hexane-AcOEt (3: 7 v/v), was subjected to reverse phase column chromatography (Cosmosil 75C18-OPN, Nacalai Tesque) using CH_3_CN–H_2_O as the eluting solvent, gel filtration (Sephadex LH-20 Lab Paks, GE Healthcare) using MeOH as the eluting solvent and HPLC (TOSOH CCPE HPLC Pump, Tosoh Corp.) that was equipped with a reverse phase column (Cosmosil 20x250 mm 5C18-Ms-II, Nacalai Tesque). The eluent was monitored at 220 nm using a Tosoh UV-8011 Tunable Absorbance Detector (Tosoh Corp.). Chromatographs were recorded using a Chromato-Pro PC integrator (Run Time Co. Ltd.) to give RA (131 mg) as an amorphous solid: [α]_D_^26^ +161.9^°^ (*c* 0.42, MeOH) IR (solid) ν_max_: 3330 (OH stretch), 1685 (C=O stretch) cm^-1^. ^1^H-NMR (500 MHz, CD_3_OD) δ 7.55 (d, 1H, *J* = 16.0 Hz, H-7), 7.03 (d, 1H, *J* = 1.8 Hz, H-2), 6.94 (dd, 1H, *J* = 1.7, 8.0 Hz, H-6), 6.77 (d, 1H, *J* = 8.0 Hz, H-5), 6.74 (d, 1H, *J* = 1.8 Hz, H-2’), 6.68 (d, 1H, *J* = 8.0 Hz, H-5’), 6.60 (dd, 1H, *J* = 1.7, 8.0 Hz, H-6’), 6.26 (d, 1H, *J* = 16.0 Hz, H-8), 5.17 (dd, 1H, *J* = 4.0, 8.5 Hz, H-8’), 3.09 (dd, 1H, *J* = 4.0, 14.3 Hz, H-7’a), 3.00 (dd, 1H, *J* = 8.5, 14.3 Hz, H-7’b). ^13^C-NMR (125 MHz, CD_3_OD) δ: 173.67 (s, C-9’), 168.46 (s, C-9), 149.60 (s, C-4), 147.68 (d, C-7), 146.66 (s, C-3), 146.00 (s, C-3’), 145.12 (s, C-4’), 129.22 (s, C-1’), 127.55 (s, C-1), 123.15 (d, C-6), 121.79 (d, C-6’), 117.51 (d, C-2’), 116.43 (d, C-5), 116.23 (d, C-5’), 115.15 (d, C-2), 114.29 (d, C-8), 74.66 (d, C-8’), 37.81 (t, C-7’). FAB-MS (positive, NBA) *m*/*z*: 383 (M^+^+Na), 361 (M^+^+H), 180 (caffeic acid), 163 (100%, caffeic acid-H_2_O). High-resolution FAB-MS calcd for C_18_H_17_O_8_: 361.0923. Found: 361.0920.

## References

[1] Koo EH, Lansbury PT Jr., Kelly JW (1999) Amyloid diseases: abnormal protein aggregation in neurodegeneration. Proc Natl Acad Sci U S A 96: 9989-9990. doi:10.1073/pnas.96.18.9989. PubMed: 10468546. 10468546 10.1073/pnas.96.18.9989 PMC33726

[2] Hardy J, Selkoe DJ (2002) The amyloid hypothesis of Alzheimer’s disease: progress and problems on the road to therapeutics. Science 297: 353-356. doi:10.1126/science.1072994. PubMed: 12130773. 12130773 10.1126/science.1072994

[3] Naiki H, Higuchi K, Hosokawa M, Takeda T (1989) Fluorometric determination of amyloid fibrils in vitro using the fluorescent dye, thioflavin T1. Anal Biochem 177: 244-249. doi:10.1016/0003-2697(89)90046-8. PubMed: 2729542. 2729542 10.1016/0003-2697(89)90046-8

[4] LeVine H 3rd (1993) Thioflavine T interaction with synthetic Alzheimer’s disease beta-amyloid peptides: detection of amyloid aggregation in solution. Protein Sci 2: 404-410. PubMed: 8453378. 8453378 10.1002/pro.5560020312 PMC2142377

[5] Klunk WE, Jacob RF, Mason RP (1999) Quantifying amyloid beta-peptide (Abeta) aggregation using the Congo red-Abeta (CR-abeta) spectrophotometric assay. Anal Biochem 266: 66-76. doi:10.1006/abio.1998.2933. PubMed: 9887214. 9887214 10.1006/abio.1998.2933

[6] Kirschner DA, Inouye H, Duffy LK, Sinclair A, Lind M et al. (1987) Synthetic peptide homologous to beta protein from Alzheimer disease forms amyloid-like fibrils in vitro. Proc Natl Acad Sci U S A 84: 6953-6957. doi:10.1073/pnas.84.19.6953. PubMed: 3477820. 3477820 10.1073/pnas.84.19.6953 PMC299203

[7] Janciauskiene S, García de Frutos P, Carlemalm E, Dahlbäck B, Eriksson S (1995) Inhibition of Alzheimer beta-peptide fibril formation by serum amyloid P component. J Biol Chem 270: 26041-26044. doi:10.1074/jbc.270.44.26041. PubMed: 7592799. 7592799 10.1074/jbc.270.44.26041

[8] Ono K, Yoshiike Y, Takashima A, Hasegawa K, Naiki H et al. (2003) Potent anti-amyloidogenic and fibril-destabilizing effects of polyphenols in vitro: implications for the prevention and therapeutics of Alzheimer’s disease. J Neurochem 87: 172-181. doi:10.1046/j.1474-1644.2003.2175_17.x. PubMed: 12969264. 12969264 10.1046/j.1471-4159.2003.01976.x

[9] Stine WB Jr., Snyder SW, Ladror US, Wade WS, Miller MF et al. (1996) The nanometer-scale structure of amyloid-beta visualized by atomic force microscopy. J Protein Chem 15: 193-203. doi:10.1007/BF01887400. PubMed: 8924204. 8924204 10.1007/BF01887400

[10] Legleiter J, Czilli DL, Gitter B, DeMattos RB, Holtzman DM et al. (2004) Effect of different anti-Abeta antibodies on Abeta fibrillogenesis as assessed by atomic force microscopy. J Mol Biol 335: 997-1006. doi:10.1016/j.jmb.2003.11.019. PubMed: 14698294. 14698294 10.1016/j.jmb.2003.11.019

[11] Jameson LP, Smith NW, Dzyuba SV (2012) Dye-binding assays for evaluation of the effects of small molecule inhibitors on amyloid (abeta) self-assembly. Acs Chem Neurosci 3: 807-819. doi:10.1021/cn300076x. PubMed: 23173064. 23173064 10.1021/cn300076x PMC3503347

[12] Tokuraku K, Marquardt M, Ikezu T (2009) Real-time imaging and quantification of amyloid-beta peptide aggregates by novel quantum-dot nanoprobes. PLOS ONE 4: e8492. doi:10.1371/journal.pone.0008492. PubMed: 20041162. 20041162 10.1371/journal.pone.0008492 PMC2794548

[13] Jarrett JT, Berger EP, Lansbury PT Jr. (1993) The carboxy terminus of the beta amyloid protein is critical for the seeding of amyloid formation: implications for the pathogenesis of Alzheimer’s disease. Biochemistry 32: 4693-4697. doi:10.1021/bi00069a001. PubMed: 8490014. 8490014 10.1021/bi00069a001

[14] Kapoor S, Priyadarsini KI (2001) Protection of radiation-induced protein damage by curcumin. Biophys Chem 92: 119-126. doi:10.1016/S0301-4622(01)00188-0. PubMed: 11527584. 11527584 10.1016/s0301-4622(01)00188-0

[15] Masuda M, Suzuki N, Taniguchi S, Oikawa T, Nonaka T et al. (2006) Small molecule inhibitors of alpha-synuclein filament assembly. Biochemistry 45: 6085-6094. doi:10.1021/bi0600749. PubMed: 16681381. 16681381 10.1021/bi0600749

[16] Ono K, Hasegawa K, Naiki H, Yamada M (2004) Curcumin has potent anti-amyloidogenic effects for Alzheimer’s beta-amyloid fibrils in vitro. J Neurosci Res 75: 742-750. doi:10.1002/jnr.20025. PubMed: 14994335. 14994335 10.1002/jnr.20025

[17] Hamaguchi T, Ono K, Murase A, Yamada M (2009) Phenolic compounds prevent Alzheimer’s pathology through different effects on the amyloid-beta aggregation pathway. Am J Pathol 175: 2557-2565. doi:10.2353/ajpath.2009.090417. PubMed: 19893028. 19893028 10.2353/ajpath.2009.090417 PMC2789642

[18] Ono K, Li L, Takamura Y, Yoshiike Y, Zhu L et al. (2012) Phenolic compounds prevent amyloid beta-protein oligomerization and synaptic dysfunction by site-specific binding. J Biol Chem 287: 14631-14643. doi:10.1074/jbc.M111.325456. PubMed: 22393064. 22393064 10.1074/jbc.M111.325456 PMC3340280

[19] Perry EK, Pickering AT, Wang WW, Houghton PJ, Perry NS (1999) Medicinal plants and Alzheimer’s disease: from ethnobotany to phytotherapy. J Pharm Pharmacol 51: 527-534. doi:10.1211/0022357991772808. PubMed: 10411211. 10411211 10.1211/0022357991772808

[20] Lamaison JL, Petitjean-Freytet C, Carnat A (1990) Rosmarinic acid, total hydroxycinnamic derivatives and antioxidant activity of Apiaceae, Borraginaceae and Lamiceae medicinals. Ann Pharm Fr 48: 103-108. PubMed: 2291599. 2291599

[21] Ono K, Hasegawa K, Naiki H, Yamada M (2004) Anti-amyloidogenic activity of tannic acid and its activity to destabilize Alzheimer’s beta-amyloid fibrils in vitro. Biochim Biophys Acta 1690: 193-202. doi:10.1016/j.bbadis.2004.06.008. PubMed: 15511626. 15511626 10.1016/j.bbadis.2004.06.008

[22] Saito T, Suemoto T, Brouwers N, Sleegers K, Funamoto S et al. (2011) Potent amyloidogenicity and pathogenicity of Abeta43. Nat Neurosci 14: 1023-1032. doi:10.1038/nn.2858. PubMed: 21725313. 21725313 10.1038/nn.2858

[23] Lesné S, Koh MT, Kotilinek L, Kayed R, Glabe CG et al. (2006) A specific amyloid-beta protein assembly in the brain impairs memory. Nature 440: 352-357. doi:10.1038/nature04533. PubMed: 16541076. 16541076 10.1038/nature04533

[24] Shankar GM, Li S, Mehta TH, Garcia-Munoz A, Shepardson NE et al. (2008) Amyloid-beta protein dimers isolated directly from Alzheimer’s brains impair synaptic plasticity and memory. Nat Med 14: 837-842. doi:10.1038/nm1782. PubMed: 18568035. 18568035 10.1038/nm1782 PMC2772133

[25] THE-ANGIOSPERM-PHYLOGENY-GROUP (2009) An update of the Angiosperm Phylogeny Group classification for the orders and families of flowering plants: APG Bot: III J Linn Soc 161: 105-121

